# Development of an AP-FRET Based Analysis for Characterizing RNA-Protein Interactions in Myotonic Dystrophy (DM1)

**DOI:** 10.1371/journal.pone.0095957

**Published:** 2014-04-29

**Authors:** Shagufta Rehman, Jordan T. Gladman, Ammasi Periasamy, Yuansheng Sun, Mani S. Mahadevan

**Affiliations:** 1 Department of Pathology, University of Virginia, Charlottesville, Virginia, United States of America; 2 Department of Biology, University of Virginia, Charlottesville, Virginia, United States of America; University of Valencia, Spain

## Abstract

Förster Resonance Energy Transfer (FRET) microscopy is a powerful tool used to identify molecular interactions in live or fixed cells using a non-radiative transfer of energy from a donor fluorophore in the excited state to an acceptor fluorophore in close proximity. FRET can be a very sensitive tool to study protein-protein and/or protein-nucleic acids interactions. RNA toxicity is implicated in a number of disorders; especially those associated with expanded repeat sequences, such as myotonic dystrophy. Myotonic dystrophy (DM1) is caused by a (CTG)_n_ repeat expansion in the 3′ UTR of the *DMPK* gene which results in nuclear retention of mutant *DMPK* transcripts in RNA foci. This results in toxic gain-of-function effects mediated through altered functions of RNA-binding proteins (e.g. MBNL1, hnRNPH, CUGBP1). In this study we demonstrate the potential of a new acceptor photobleaching assay to measure FRET (AP-FRET) between RNA and protein. We chose to focus on the interaction between MBNL1 and mutant *DMPK* mRNA in cells from DM1 patients due to the strong microscopic evidence of their co-localization. Using this technique we have direct evidence of intracellular interaction between MBNL1 and the *DMPK* RNA. Furthermore using the AP-FRET assay and MBNL1 mutants, we show that all four zinc-finger motifs in MBNL1 are crucial for MBNL1-RNA foci interactions. The data derived using this new assay provides compelling evidence for the interaction between RNA binding proteins and RNA foci, and mechanistic insights into MBNL1-RNA foci interaction demonstrating the power of AP-FRET in examining RNA-Protein interactions in DM1.

## Introduction

Förster Resonance Energy Transfer (FRET) microscopy is a powerful tool widely used to identify molecular interactions in live or fixed cells. FRET is a non-radiative transfer of energy from a donor fluorophore in the excited state to an acceptor fluorophore in close proximity [1]–[3]. Since the efficiency of energy transfer (E%) varies inversely with the sixth power of the intermolecular distance, the distance over which FRET can occur is limited to 1–10 nm [1]–[3], making FRET a powerful technique in identifying molecular interactions [4].

Myotonic Dystrophy type 1 (DM1), a dominantly inherited multisystemic neuromuscular disorder is the first example of RNA-mediated disease amongst genetic disorders [5], [6]. DM1 is caused by a CTG repeat expansion in the 3′ untranslated region (3′ UTR) of the *DMPK* gene [7], [8]. As a result, mutant *DMPK* mRNA is retained in the nucleus as discrete foci, or RNA foci [9]. These RNA foci differ in their shape, size and cellular abundance [10]. Little is known about the composition of RNA foci as there is no method available to purify the foci intact, and nothing is known about RNA-protein and protein-protein interactions at RNA foci in DM1. In DM1, the functions of RNA binding proteins like muscleblind-like protein 1 (MBNL1) and CUG-binding protein-1 (CUGBP1), which are developmental regulators of alternative splicing, are affected resulting in numerous splicing abnormalities [11]–[17]. CUGBP1 levels are elevated in DM1 whereas functional levels of MBNL1 are thought to be depleted due to its sequestration by mutant RNA foci. Though co-localization of MBNL1 with the mutant RNA foci in different DM1 tissues and models of RNA toxicity has been previously demonstrated there is no direct evidence of intracellular interaction [18]–[23].

In this study, we have developed and used an acceptor photobleaching FRET assay to identify RNA-protein interactions. Using this technique we provide the first direct evidence of intracellular interaction between endogenous MBNL1 and mutant *DMPK* mRNA foci in cells derived from DM1 patients. We have corroborated our findings with EGFP-fused MBNL1 and have used RNA-IP with anti-MBNL1 antibodies to biochemically validate the FRET analysis. Further, we have used deletion mutagenesis to provide mechanistic insights by identifying functional domains in MBNL1 involved in this interaction and in regulation of alternative splicing. Taken together these results demonstrate the power of AP-FRET in not only identifying interactions between RNA and proteins but also in determining the functional domains involved in that interaction.

## Material and Methods

### RNA FISH and immunofluorescence workflow

DM1 cells were grown on a glass coverslip. When the desired cell density was reached the cells were washed in PBS three times then fixed in 4% paraformaldehyde/PBS for 10 min at room temperature. Following fixation they were permeabilized in cold 2% acetone/PBS for 5 min at room temperature. The cells were then washed with PBS three times and incubated with 30% formamide/2x SSC buffer at 37°C for 10 min. Hybridization was then carried out with either a CY3 or FITC labeled (CAG)_10_ probe at 0.1 ng/uL for 2 h at 37°C in the hybridization buffer (30% formamide, 2x SCC, 0.02% BSA, 66 ug/mL yeast tRNA, 2 mM vanadyl complex). After the hybridization, the cells were washed in 30% formamide/2xSSC at 45°C for 30 min. Next the cells were blocked in 1% BSA/PBS for 1 h at 37°C. Primary antibody, either MBNL1 rabbit polyclonal antibody (1∶2000) [22], RNA Pol II rabbit polyclonal antibody (1∶100) (SCBT, USA) or hnRNPH goat polyclonal antibody (1∶200) (SCBT, USA) was used in 1%BSA/PBS for 1 h at 37°C. After primary incubation the cells were washed in PBS three times for 10 min each. The cells were then labeled in the appropriate secondary antibody, Alexa488 or Alexa555 (1∶500) (Molecular Probes, USA) in 1%BSA/PBS for 1 h at 37°C. The cells were washed three more times in PBS for 10 min each then mounted in Vectashield (Molecular Probes). This protocol was used for all assays.

### AP-FRET

Acceptor photobleaching FRET (AP-FRET) was performed on a Leica SP5X white light laser (WLL) confocal microscope using the Leica AP-FRET wizard. Images were acquired using a 63x NA 1.4 oil objective on a 512x512 resolution format. In order to minimize photobleaching during image acquisition, low percentages (5–25%) of the full power of WLL 488 nm and 552 nm with PMT gain of 650 v were used. A region of interest (ROI) was drawn over the selected foci and either 100% of WLL 552 nm for 8 iterations was used for photobleaching the acceptor/CY3-RNA foci or 40% of WLL 552 nm for 6 iterations for photobleaching acceptor/Alexa555-MBNL1 or RNA Pol II to background levels. The “n” in the figure legend denotes number of RNA foci (ROIs) analyzed in DM1 cells. Prebleach and Postbleach images were acquired using identical imaging settings. A 2x2 pixel ROI was drawn over bleached foci and efficiency of energy transfer (E%) was calculated using the equation: E%  =  (Donor Postbleach-Donor Prebleach)* 100/(Donor Postbleach). Representative images were processed using Adobe Photoshop CS3 and Minitab 16 was used for E% distribution graphs.

### RNA-Immunoprecipitation (RNA-IP)

DM200 mouse skeletal muscle [16] and DM1 primary fibroblasts with a BpmI polymorphism in exon 10 of the *DMPK* gene [24] were used for RNA-IP with a MBNL1 monoclonal antibody (Abnova, Taiwan) without any cross-linking agents. For DM1 fibroblasts, 1.5x10^6^ cells were trypsinized, pelleted, PBS washed and processed for nuclear-cytoplasmic fractionation using the PARIS kit (Ambion, USA). The nuclear lysate was diluted 1∶3 in Sanford IP-RT PCR buffer (10 mM HEPES pH 7.3, 100 mM NaCl, 3 mM MgCl2, 0.5% NP-40, 0.5% Triton X-100) supplemented with, RNase inhibitor to a final concentration of 100 units/mL, Protease inhibitor to a final concentration of 1x, Phosphatase inhibitor to a final concentration of 1x, and DTT to a final concentration of 1 mM. The DM1 fibroblasts' nuclear lysate and DM200 skeletal muscle homogenate were immunoprecipitated either with anti-MBNL1 or isotype control using magnetic beads (GE Healthcare, United Kingdom). RNA was extracted with Trizol (Invitrogen, USA), followed by EGFP RT-PCR for the mutant transgene and *DMPK* RT-PCR/BpmI RFLP for mutant and wildtype transcripts. *DMPK* RT-PCR primer for cDNA synthesis: 5′ ACT GGA GCT GGG CGG AGA CCC A 3′ and *DMPK* RT-PCR primers: FP- 5′ GGCTCACTGCCATGGTGAG 3′, RP- 5′ CTCGGCCTCAGCCTCTGC 3′.

### Generation of MBNL1 deletion mutants

The pEGFP-*MBNL1* backbone was used for generating the MBNL1 deletion mutants. The Quick exchange mutagenesis kit (Agilent Technologies, USA) was used for generating mutants: Del-C′, Del-Linker and Del-ZF4-C′; whereas, a Pst1 restriction site within the *MBNL1* cDNA was used for generating Del-N′ followed by conventional self ligation and transformation. All deletion mutants were sequence confirmed and were in frame with the N′ terminus EGFP tag. Primers for quick exchange mutagenesis are as follows. Primer set 1: FP-Del-C′: 5′ gccaccgcagctgccatgtagccacaagtatggatcc 3′; RP-Del- C′: 5′ ggatccatacttgtggctacatggcagctgcggtggc 3′. Primer set 2: FP-Del-ZF4-C′: 5′ gcacaatgattgacaccaattagccacaagtatggatcc 3′; RP-Del- ZF4-C′: **5**′ ggatccatacttgtggctaattggtgtcaatcattgtgc 3′. Primer set 3: FP-Del-Linker: 5′ gccccattacaacccgtgacagacagacttgaggtatg 3′; RP-Del-Linker: 5′ catacctcaagtctgtctgtcacgggttgtaatggggc 3′

### Splicing assay

Total RNA was extracted using the lysis buffer (2% SDS, 500 mM NaCl,10 mM Tris-HCl pH 7.2, 1.5 mM MgCl2, 10 mM EDTA pH 8.0, Prot K 0.5 mg/mL in RNAse free water [25] and subsequent extraction using Trizol. The cDNA synthesis was done using the Quantitect kit (Qiagen, Netherlands) followed by RT-PCR for endogenous *SERCA1* exon 22 with primers forward: 5′ ATCTTCAAGCTCCGGGCCCT 3′ and reverse: 5′ CAGCTCTGCCTGAAGATGTG 3′; *IR* exon 11 with primers forward: 5′ CCAAAGACAGACTCTCAGAT 3′ and reverse: 5′ AACATCGCCAAGGGACCTGC 3′ and *NFIX* exon 7 with primers forward: 5′ AGCCCTGTTGATGACGTGTT 3′ and reverse: 5′ AGTGCAGGGCTGATGCTGT 3′. Minitab 16 was used for one-way ANOVA with Tukey's multiple comparison method for statistical significance.

### Cell culture

The immortalized DM1 fibroblasts/myoblasts inducible for the *MYOD* gene were obtained from Genomics Institute of the Novartis Research Foundation, CA [26]. They were immortalized by constitutive overexpression of human telomerase reverse transcriptase (hTERT) gene and were cultured at 37°C in DMEM supplemented with 15% FBS and 1x penicillin-streptomycin (Invitrogen, USA). The DM1 primary fibroblasts with BpmI polymorphism were a gift from Dr. David Brook [24] and were cultured in 20% FBS with 1x penicillin-streptomycin (Invitrogen, USA). HEK293T cells were grown at 37°C in DMEM supplemented with 10% FBS and 1x penicillin-streptomycin (Invitrogen, USA). The CTG repeat length in the *DMPK* gene of the immortalized DM1 fibroblasts used for the AP-FRET and splicing assay and the primary DM1 fibroblasts used for the RNA-IP is (CTG)_1000_.

### Transient transfections

Transient transfections in DM1 fibroblasts/myoblasts were done using NucleofectorTM (Amaxa, Germany), and in HEK293T using Lipofectamine 2000 (Invitrogen, USA). Cells were either fixed for RNA FISH or processed for RNA extraction 48h post transfection or 24 h for HEK293T. For AP-FRET experiments (1 µg) and for splicing assays (4 µg) of plasmids were used. For the EGFP-tagged RNA binding proteins and for MBNL1-full length (MBNL1- FL) and its deletions, the coverslips were mounted after RNA-FISH and subsequently analyzed by AP-FRET. Plasmids pEGFP-MBNL1, pEGFP-hnRNP-C and pEGFP-CUGBP1 were gifts from Dr. David Brook.

### Western blot

Protein expression of FL-MBNL1 and its deletion mutants and their molecular sizes were analyzed and confirmed by western blotting. The blot was probed with anti-EGFP and anti-Dynein antibodies (Invitrogen, USA).

### Statistical methods

Standard statistical methods were employed using the software Minitab 16.1.0, produced by Minitab, Inc. Two-sample one-sided T-tests were used to determine differences in E% between donor-acceptor and donor only FRET. A one-sided Fisher's exact test was used to determine differences in positive E% between groups. A one-way ANOVA with Tukey's multiple comparison was used to analyze the alternative splicing results. Statistical significance was set at a p-value of <0.05.

## Results

### AP-FRET provides evidence of interaction between endogenous protein and RNA

Though conventional or laser scanning confocal microscopy (LSCM) can be used to detect the co-localization of molecules, its optical resolution (typically 200 nm) limits the ability to make any conclusive statements about molecular interactions. However, FRET microscopy typically detects interactions in the 1–10 nm range. FRET can be analyzed by a number of different methods [1], [3]. We have used FRET analysis by acceptor photobleaching (AP-FRET) as it is the most direct method of detecting and/or verifying FRET between molecules labeled with donor and acceptor fluorophores [27] without the need for extensive bleed-through corrections required in spectral and confocal FRET methods. AP-FRET is an intensity based method to study interaction and therefore, the concentrations of the donor and acceptor fluorophores, labeling method, the choice of the right FRET pair, all play a very important role. Also, of importance is the expression level of the targets and their topological arrangement in a RNA-protein complex as this will determine the labeling, the donor and acceptor stoichiometry and therefore detection of their interaction. Another key aspect to successful AP-FRET is to select an appropriate wavelength and laser intensity that selectively excites and photobleaches the acceptor, without affecting the excitation potential of the donor. In steady state when the donor is excited, FRET involves transfer of energy from a donor to an acceptor molecule resulting in decreased fluorescence (quenching) of the donor molecule. In AP-FRET, when the acceptor is specifically excited and photobleached prior to donor excitation, it is no longer capable of receiving transferred energy when the donor is excited, with the end result being increased signal from the donor molecule (dequenching) upon signal acquisition. The relative change in donor signal before and after photobleaching (termed E%) is a measure of FRET efficiency which reflects the relative proximity of the donor and acceptor molecules. To measure and rule out the background noise arising due to instrumental fluctuations and/or due to fluorophore instability and to verify that the observed E% represents a positive interaction, we also measured the distribution of E% from a donor only (no acceptor) labeled control. Positive interactions were scored as any E% over the value that excludes ∼95% of the E% measured in experimental negative control. Finally the number of positive interactions was statistically compared between the donor-acceptor labeled samples and the donor only samples. A statistical difference between the groups represent actual FRET while no statistical difference between the donor-acceptor and donor only sections represented a failure to detect an interaction either because no interaction existed or because our assay was unable to accurately identify the interaction.

**Figure 1:**
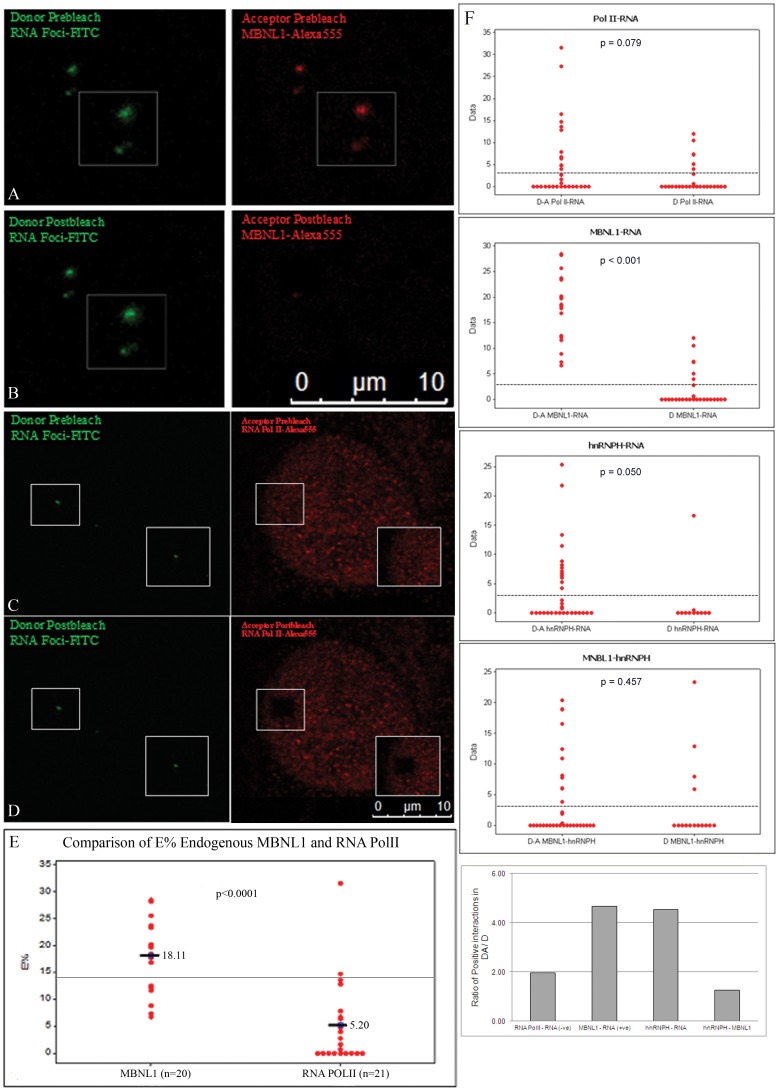
Evidence of intracellular interaction between endogenous MBNL1, and hnRNPH with RNA foci using AP-FRET. RNA foci in DM1fibroblasts were detected by RNA-FISH (green) in combination with immunofluorescence for either endogenous MBNL1 or RNA Pol II (red). FITC-Alexa555 was used as the FRET pair. Representative donor and acceptor pre-bleach (A and C) and post-bleach (B and D) images are shown for MBNL1-RNA foci or RNA Pol II foci AP-FRET. Dequenched signal from the donor (FITC) was seen after photobleaching the acceptor demonstrating interaction of MBNL1 with RNA foci. (E) Comparison of E% distribution for FRET assays of RNA foci and MBNL1 interactions in DM1 cells (n = 20) and for RNA foci and RNA Pol II interactions (n = 21). (F) Comparison of E% distribution in FRET assays for interactions of RNA Pol II, MBNL1, and hnRNPH with RNA foci, and for interactions of hnRNPH and MBNL1. A normalized ratio of the number of positive ROIs in Donor/Acceptor (DA) to positives in Donor only (D) FRET assays underscores that both hnRNPH and MBNL1 interact with the RNA foci. Line on graphs represents the positive FRET threshold level.

To demonstrate the utility of AP-FRET in identifying RNA-protein interactions we first turned to the interaction of MBNL1 and *DMPK* RNA, which have been extensively studied in myotonic dystrophy. Using fibroblasts from DM1 patients we performed a combined RNA-FISH potocol to simultaneously detect RNA foci with a FITC labeled (CAG)_10_ antisense probe (the donor), and MBNL1 (the RNA-binding protein) with an Alexa555 labeled secondary antibody (the acceptor). To optimize of photobleaching for the AP-FRET experiment we avoided any saturation in our Regions of Interest (ROIs) by using low power of the exciting laser below saturation for signal acquisition. These conditions result in images of MBNL1 foci without excessive nucleoplasmic/cytoplasmic MBNL1 staining. After optimizing photobleaching conditions, AP-FRET was performed using a LSCM (Leica SP5X white light laser (WLL)). Dequenched signal from the donor was seen after photobleaching the acceptor, demonstrating FRET and an interaction between MBNL1 and RNA foci (Fig. 1A–B). The specificity of the AP-FRET assay was established using RNA polymerase II (RNA Pol II) as a negative control (Fig. 1C–D). Prebleach and Postbleach images were used to calculate an average E% of 18.11% for MBNL1-RNA foci interactions as compared to a value of only 5.20% for RNA Pol II (Fig. 1E). The variability in E% distribution between various regions of interest (ROIs) is likely the result of observed variability in intensity of RNA foci and MBNL1 aggregates or differences in donor/acceptor stoichiometry at a particular RNA focus, however it was clear that MBNL1 interactions were significantly higher than the donor alone controls. In contrast the RNA Pol II was indistinguishable from its donor only samples verifying it as an appropriate negative control. These results provide the first compelling evidence for an intracellular interaction between MBNL1 and the mutant RNA foci in cells from DM1 patients.

**Figure 2:**
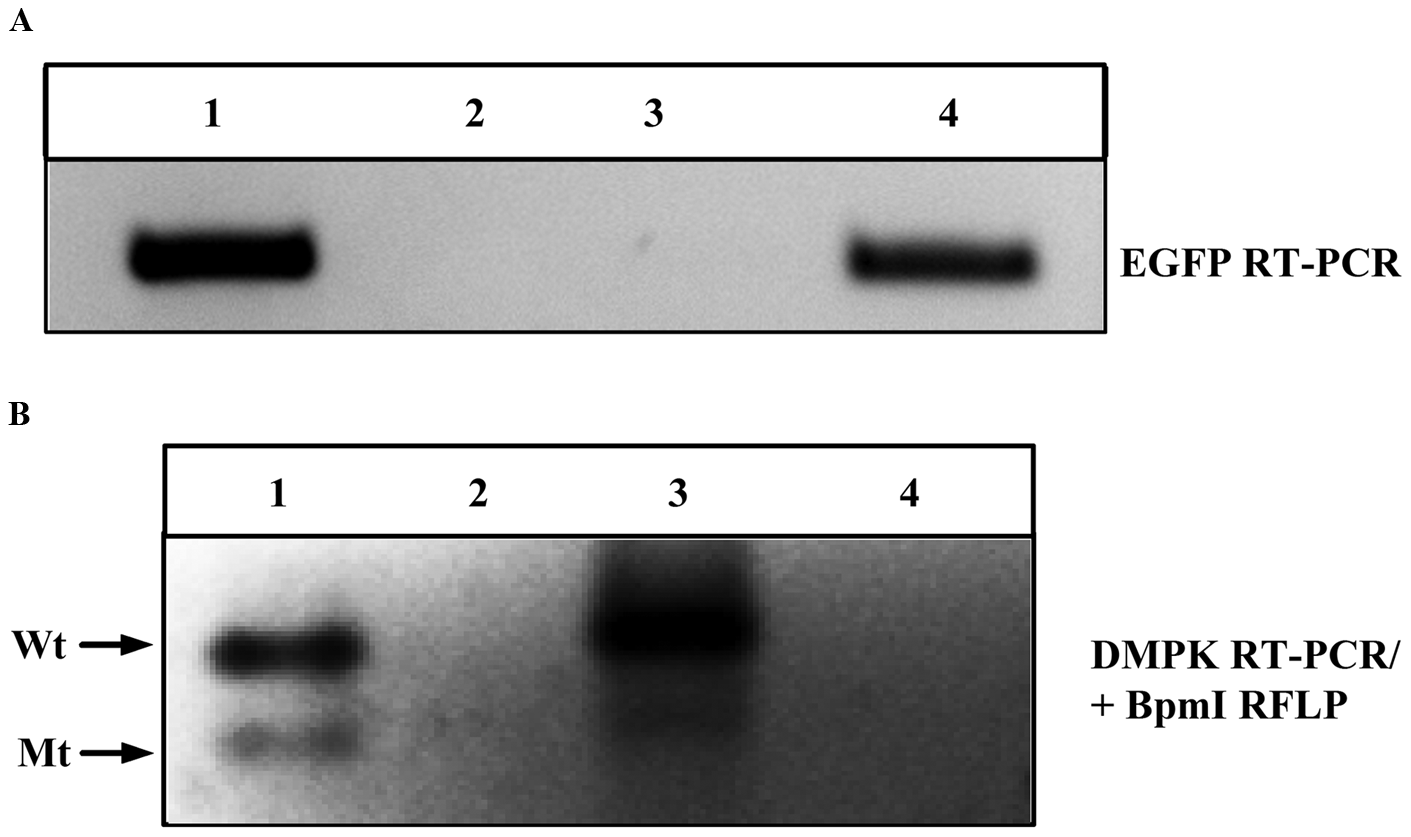
RNA-Immunoprecipitation shows association of mutant *DMPK* transcripts with MBNL1. RNA-IP using MBNL1 monoclonal antibodies detect (A) mutant transgene/EGFP mRNA from DM200 skeletal muscles (lane 4) and (B) mutant (Mt) as well as wildtype (Wt) *DMPK* mRNA from DM1 fibroblasts (lane 3). Isotype control antibodies (lane 2) or beads only (lanes A3 and B4) do not bring down eGFP or *DMPK* mRNAs. Lane 1 is the input.

While the interaction of MBNL1 and the mutant *DMPK* RNA has been extensively studied we choose to carry out RNA-IP to detect *DMPK* transcripts binding to MBNL1 as a way to confirm our assay. We used skeletal muscle tissue from a mouse model expressing a EGFP-*DMPK* 3′ UTR (CTG)_>200_ transgene (termed DM200) [16]. In addition we used DM1 fibroblasts which are polymorphic for a BpmI restriction site in exon 10 of the *DMPK* gene [24]. This polymorphism enabled us to distinguish between mutant and wildtype transcripts by BpmI restriction fragment length polymorphism (RFLP). Since the RNA foci are nuclear, we utilized fractionated nuclear RNA extracts from the DM1 fibroblasts, and total RNA extracts from the DM200 skeletal muscles. RNA-IP with anti-MBNL1 monoclonal antibodies was performed. The immunoprecipitated RNA was extracted and analyzed by RT-PCR for the presence of the EGFP transcript in the mouse extracts (Fig. 2A), or by RT-PCR/BpmI RLFP for the detection of *DMPK* transcripts in DM1 fibroblast extracts (Fig. 2B). Both of these assays clearly show immunoprecipitation of either the EGFP mRNA or the mutant *DMPK* mRNA by anti-MBNL1 but not by the isotype control antibodies. This provided confirmatory biochemical evidence for the interaction between MBNL1 and the mutant *DMPK* transcript in both human cells and mouse tissues.

We next looked at another RNA binding factor with much less evidence for RNA foci binding, hnRNPH, using our AP-FRET assay. There is evidence that hnRNPH is both increased in DM1 but also is pulled down with a modified UV crosslinking assay using *DMPK* derived RNA [28], [29]. Again using fibroblasts from DM1 patients we performed a combined RNA-FISH/immunofluoresence protocol to simultaneously detect RNA foci with a FITC labeled (CAG)10 antisense probe (the donor), and hnRNPH (the RNA-binding protein) with an Alexa555 labeled secondary antibody (the acceptor). After optimizing photobleaching conditions, AP-FRET was performed again using the protocols designed to avoid any saturation in our Regions of Interest (ROIs). The E% was calculated and the number of positive interactions from the donor-acceptor labeled samples was compared to the donor only samples for hnRNPH. We again used FRET results from RNA polymerase as our negative control and MBNL1 as our positive control (Fig. 1F). There was a measurable difference between the E% from the donor-acceptor FRET for hnRNPH compared to the donor only control (Fig. 1F). We observed dequenched signal from the donor after photobleaching of the acceptor at many but not all of the RNA foci (14/34), demonstrating FRET and intracellular interaction between hnRNPH and RNA foci at these RNA foci. To normalize the data and compare MBNL1, RNA Poll II, and hnRNPH we took the proportion of positive interactions measured in the donor-acceptor group and divided it by the proportion of positive interactions measured in the donor only group. When completed we saw a similar ratio in the MBNL1 group (our positive control) and hnRNPH. However, unlike the MBNL1-RNA interactions taking place at all the foci examined (21/21) this more varied pattern of binding may represent a less frequent or transient interaction at the foci. Though unlikely it could also be possible that hnRNPH indirectly interacted with the RNA foci through another protein, such as MBNL1. To address this possibility we measured the E% distribution between hnRNPH and MBNL1 and found no evidence of interaction (Fig. 1F). Using the AP-FRET assay we were able to provide evidence of interaction between endogenous proteins (MBNL1 and hnRNPH) and RNA (*DMPK* expanded repeat transcripts).

### AP-FRET for studying intracellular interaction between RNA foci and fluorescent fusion RNA-binding proteins

Identifying and characterizing RNA-protein interaction can be a difficult undertaking, especially as many techniques to study the process do not provide evidence of direct interaction. FRET is a distance dependent phenomenon [1]–[3], which allows direct testing of an RNA and protein interaction. However with the use of primary and secondary antibodies, as was done in the previous experiments, steric hindrance could result in no or lower FRET E%. The reporter EGFP is a small protein (∼27 kDa) and the chances of steric hindrance may be less with its use. Therefore, we used EGFP fused MBNL1 (EGFP-MBNL1) to independently detect intracellular interactions and study the possibility of RNA-protein interactions by other RNA binding proteins in DM1.

**Figure 3:**
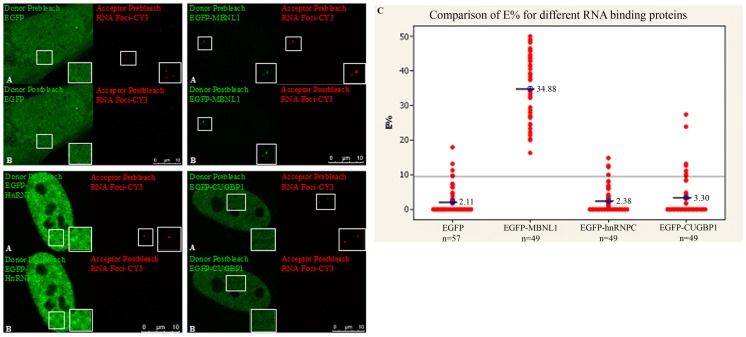
Demonstration of intracellular interaction between RNA foci and EGFP-MBNL1 using AP-FRET. DM1 fibroblasts were transfected with plasmids encoding EGFP-MBNL1, EGFP-hnRNPC, EGFP-CUGBP1 and EGFP alone. RNA-FISH was carried out 48 h post-transfection with CY3-(CAG)_10_ antisense probe. EGFP-CY3 was used as FRET pair. Representative donor and acceptor (A) pre-bleach and (B) post-bleach images for each experiment are shown. Strong dequenched signal from the donor could be seen only for EGFP-MBNL1 after photobleaching the acceptor. (C) E% distribution for different ROIs shows E% for EGFP-MBNL1 with an average of 34.8% (n = 49) and lower or background level E% distribution for EGFP-hnRNPC (n = 49) and EGFP-CUGBP1 (n = 49) similar to EGFP alone (n = 57). Line on graphs represents the positive FRET threshold level.

EGFP-MBNL1 corresponds to an isoform of MBNL1 (expressing exons 1–4, 6 and 10) that has previously been shown to co-localize with the RNA foci [19]. Plasmid expressing EGFP-MBNL1 were transfected into DM1 cells and upon RNA-FISH with a CY3-(CAG)_10_ antisense probe, AP-FRET was performed using EGFP (donor) and CY3 (acceptor) as the FRET pair. As a negative control, plasmid expressing EGFP alone was used to measure background noise in AP-FRET experiments. Similar experiments were done using EGFP fused versions of CUGBP1 and hnRNP-C, two other RNA-binding proteins thought to have *in vitro* binding with the *DMPK* mRNA [12], [30]. Only EGFP-MBNL1 showed interactions with the RNA foci with an average E% of 34.88% and a positive FRET ratio of 49/49 compared to 2.11% for EGFP-alone control and a positive FRET ratio of 4/57 (Fig. 3), confirming the results obtained using antibodies against MBNL1. Notably, we did not detect interaction between RNA foci and EGFP-CUGBP1 or EGFP-hnRNP-C by AP-FRET (Fig. 3). This assay thus allows the detection of RNA binding proteins that bind to RNA, in this case MBNL1.

### AP-FRET can be used to identify domains responsible for RNA-protein interactions and function

The domains in a protein that are responsible for binding and the domains in a protein that have other biological functions can be difficult to study independent of each other. Utilizing AP-FRET in the context of the DM1 MBNL1-RNA interaction we examined the different domains present in MBNL1 and how they relate to its RNA binding and mRNA splicing activity.

**Figure 4:**
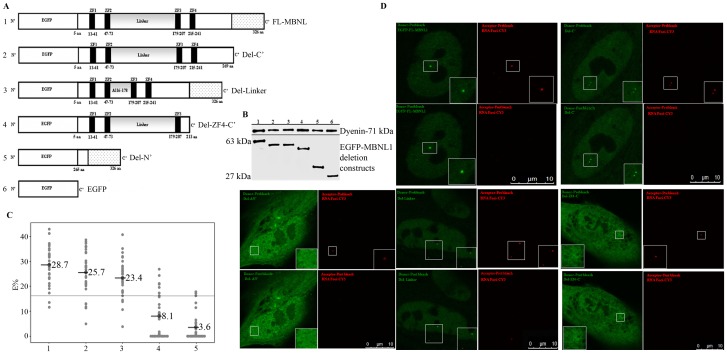
Identification of domains in MBNL1 responsible for interaction with RNA foci using AP-FRET. (A) Schematic representation of FL-MBNL1 and its deletion mutants. The FL-MBNL1 has four zinc-finger motifs (black boxes), a linker region (grey) and a C′ terminal domain (stippled). Each construct contains an N′ terminal EGFP tag. (B) Western blot of transfected EGFP-fused FL-MBNL1 and its deletion mutants in HEK293Ts; (lower panel) blot probed with antibodies for EGFP and (upper panel) probed with Dynein. (C) E% distribution for the different ROIs. Similar E% distribution was seen for FL-MBNL1 (n = 31), Del-C′ (n = 33) and Del-Linker (n = 34); Del-ZF4-C′ (n = 30) resulted in a dramatic drop in FRET efficiency values whereas Del-N′ (n = 33) showed loss of interaction. The line on the graph represents the positive FRET threshold level. (D) AP-FRET analysis of deletion mutants versus FL-MBNL1 was performed with line averaging (Line Av = 2). Representative donor and acceptor pre-bleach and post-bleach images for each experiment are shown.

MBNL1 contains two pairs of zinc-finger motifs, a linker region between zinc-finger pairs and a C′ terminal domain (Fig. 4A) [19]. A previous study using a yeast three-hybrid system showed that all four zinc-finger domains are necessary for interaction with (CUG)_21_ and (CCUG)_22_ RNAs [31]. However, no *in vivo* data exists defining the domains of MBNL1 necessary for interactions with the mutant *DMPK* mRNA. To address this we generated a series of MBNL1 deletion mutants using the EGFP-MBNL1 backbone (Fig. 4A). All of the mutants: Del-C′ (lacking the carboxy terminus), Del-Linker (lacking the linker region), Del-214-326 (lacking the fourth zinc finger and C′ terminus) and Del-N′ (lacking all four zinc finger domains and the linker) were expressed in HEK293T cells, and western blotting was used to verify their expression and appropriate size (Fig. 4B). These constructs were also transfected in DM1 fibroblasts and the cells were then analyzed by RNA-FISH and AP-FRET analysis.

The Del-C′ and Del-Linker both of which had all four intact zinc-finger motifs (ZF1-ZF4), showed strong dequenched signal from the donor after photobleaching the acceptor (Fig. 4C and 4D). Their E% distributions were similar to full length-MBNL1 (FL-MBNL1) (Fig. 4C and 4D). On the other hand, deletion of all four zinc-finger motifs (Del-N′) resulted in complete loss of interaction. Deletion of ZF4-C′ (Del-214-326) resulted in a dramatic drop in FRET efficiency values (Fig. 4C and 4D) with an average E% of 8.1% and positive FRET ratio of 8/53 as compared to 28.7% and 29/31 for FL-MBNL1 showing that loss of ZF4 had a significant negative effect on MBNL1-RNA foci interaction. These results are analogous to results using the yeast three-hybrid system where loss of binding with (CUG)_21_ RNAs was observed when ZF4, or the linker region were deleted [31]. However, we did not observe a loss of interaction with RNA foci after deletion of the linker region. Our AP-FRET analysis using MBNL1 deletion mutants underscores that all four zinc-fingers are crucial for interaction with RNA foci.

**Figure 5:**
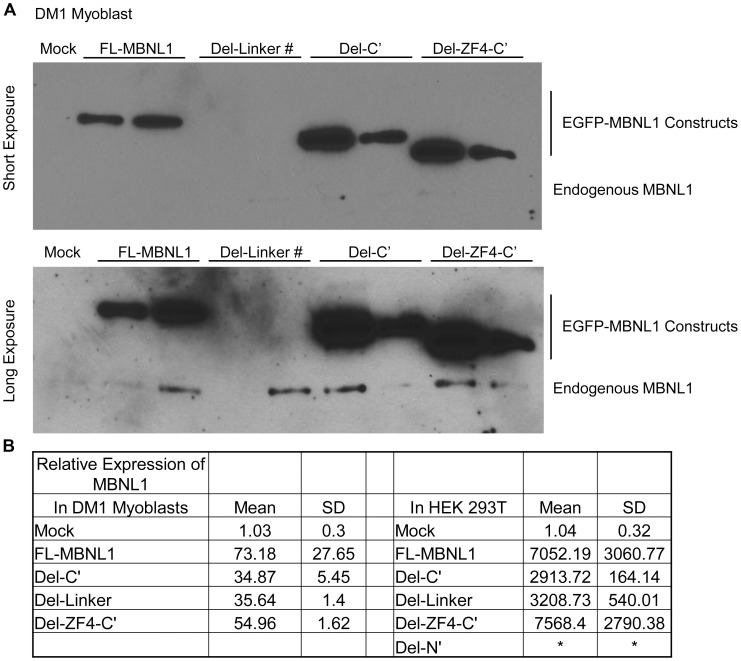
Over-expression of the MBNL1 constructs leads to robust protein and mRNA over-expression. A. Western blot of the DM1 human myoblasts transfected with various EGFP-MBNL1 fusion constructs shows significant over-expression of the fusion proteins as compared to the endogenous MBNL1. # represents the Del-Linker fusion protein which cannot be picked up by the MBNL1 antibody. B. Real Time PCR was performed to quantify total *MBNL1* levels in cells over-expressing the various EGFP-MBNL1 constructs and compared to the levels from mock transfected cells. There was extensive over-expression in these cell lines. * represents the Del-N constructs which the *MBNL1* qRTPCR primers did not recognize due to their location within the deleted region.

MBNL1 is also a known regulator of alternative splicing of a number of transcripts whose splicing is mis-regulated in DM1, including insulin receptor (*IR*), muscle-specific chloride channel (*CLCN1*) and sarcoplasmic/endoplasmic reticulum Ca^+2^ ATPase (*ATP2A1*, also known as *SERCA1*) [32]-[36]. MBNL1 in conjunction with other splicing regulators controls the transition from embryonic to adult splicing pattern [29]. Using DM1 myoblasts and HEK293T cells we assessed the role of MBNL1 deletion mutants in the regulation of alternative splicing; specifically of endogenous *SERCA1* exon 22, *IR* exon 11 and *NFIX* exon 7 [35]. HEK293T cells have a high level of CUGBP1 and relatively low level of MBNL1, analogous to the DM1 condition, and express an embryonic pattern of splicing with a predominance of *SERCA1* without exon 22, *IR* without exon 11 and *NFIX* with exon 7. Western bloting and qPCR were used to verify that the transfected cells were over-expressing the relevant MBNL1 constructs (Fig. 5).

**Figure 6:**
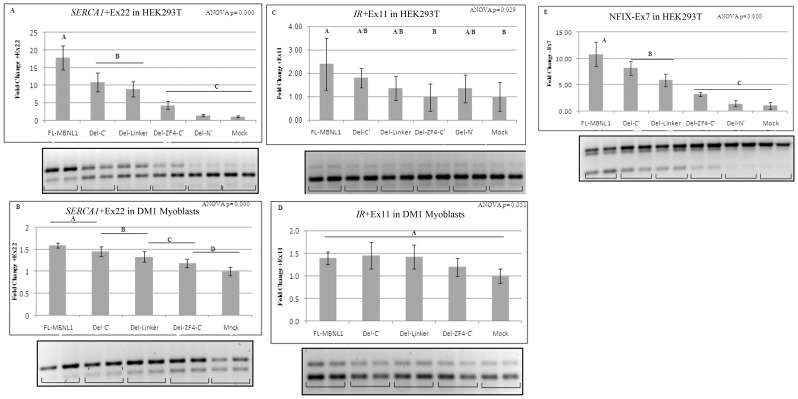
Identification of domains in MBNL1 responsible for regulation of alternative splicing of endogenous (A–B) *SERCA1* exon 22, (C–D) *IR* exon 11 and (E) *NFIX* exon 7. RT PCR splicing assay results for inclusion/exclusion levels of respective exons upon over-expression of FL-MBNL1, its deletion mutants or empty vector/Mock in (A,C,E) HEK293T and (B and D) DM1 myoblasts. At least four independent transfections were done for each construct. Relative fold change compared to Mock for inclusion/exclusion levels of respective exons is shown. Error bars denote standard deviation (SD) on the graphs. One-way ANOVA with Tukey's multiple comparison method for statistical significance was performed. Means that do not share a letter are significantly different.

RT-PCR showed that over-expression of FL-MBNL1 resulted in 17.82 fold increase in *SERCA1* exon 22 inclusion (Fig. 6A) and 10.79 fold increase in *NFIX* exon 7 exclusion (Fig. 6E). The deletion mutants of MBNL1 showed progressive loss of positive splicing regulation for *SERCA1* exon 22 and *NFIX* exon 7: with the Del-C′ showing a 40% reduction, Del-Linker a 51% reduction in splicing regulation for *SERCA1* exon 22; and Del-C′ showing a 25% reduction and Del-Linker a 46% reduction in splicing regulation for *NFIX* exon 7 as compared to FL-MBNL1; Del-N′ showed almost complete loss of splicing regulation (Fig. 6A and 6E). A similar incremental loss in positive regulation was observed in DM1 myoblasts for *SERCA1* exon 22 (Fig. 6B). The *NFIX* defect was absent in DM1 myoblasts. Deletion of ZF4 (Del-214–326), resulted in significant loss in splicing regulation in both HEK293T and DM1 myoblasts for endogenous *SERCA1* and *NFIX*, analogous to what was observed using *IR* and *cTNT* minigenes in COSM6 cells [37].

Splicing assay results for endogenous *IR* showed that over-expression of FL-MBNL1 resulted in 2.4 fold increase in *IR* exon 11 inclusion in HEK293T (Fig. 6C) and deletion of ZF4 (Del-214–326) resulted in significant loss in splicing regulation. However, for the other deletion mutants the data was not statistically different. Also, the splicing data on endogenous *IR* exon 11 inclusion in DM1 myoblasts for the FL-MBNL1 and its deletion mutants was not statistically different (Fig. 6D).

## Discussion

Regulation of RNA processing, stability, and localization are essential for the proper functioning of the cell. Many of these functions such as mRNA splicing, microRNA processing, RNA shuttling, and RNA sequestration are regulated by RNA-protein interactions. Biochemical studies aimed at understanding RNA-protein interaction are limited by *in vitro* conditions while techniques like immunofluorescence can only suggest co-localization not actual interaction. When an aspect of normal RNA regulation is defective numerous diseases can arise. This is especially true in many of the expanded RNA repeats disease, of which DM1 is a member. We developed a technique combining RNA-FISH and AP-FRET to study the function and interactions of RNA and proteins. We have demonstrated the utility of this assay by studying the well characterized interaction of MBNL1 with the toxic RNA foci that occur in DM1 and then continued to study other RNA binding proteins such as hnRNPH, CUGBP1, and hnRNPC.

The main pathogenic process in DM1 is described as the nuclear retention of mutant *DMPK* transcripts into discrete RNA foci which are thought to be deleterious due to their interactions with RNA binding proteins. The role of MBNL1 in RNA foci formation in DM1 has been implicated ever-since its identification as the “EXP” protein in 2000 [11]. Since then, MBNL1 has been a subject of intense study using various models for DM1: from human tissues [20], [21], to mice [14], [23] to flies [38], [39]. However, no studies have shown direct intracellular interaction between MBNL1 and RNA foci. The AP-FRET assay used in this study provides the first compelling evidence for this interaction between MBNL1 and RNA foci in DM1 cells (Fig. 1). Using an independent biochemical approach, RNA-IP with an anti-MBNL1 antibody clearly shows the pull down of mutant *DMPK* transcripts from extracts made from DM1 cells and from skeletal muscles of transgenic mice expressing the EGFP-*DMPK* 3′ UTR (CTG)_>200_ transgene (Fig. 2). Together, these two assays along with data generated by other groups on the co-localization, biochemical interaction, and preferred binding substrate of MBNL1 provides strong support for the physical interaction between MBNL1 and *DMPK* mRNAs.

Interestingly, the RNA-IP from DM1 cells also revealed that the normal *DMPK* transcript interacts with MBNL1. The functional relevance of this is currently unknown, but in the context of a cryptic splice site within the *DMPK* 3′ UTR [30] and a transgenic mouse model over-expressing a normal *DMPK* 3′ UTR that develops DM1 pathology [16], this is an intriguing finding. Furthermore, we took this assay and explored the less characterized but previously identified potential interaction between hnRNPH and *DMPK* RNA foci. Using the AP-FRET assay we found evidence of an interaction between the toxic RNA foci that was independent of an interaction with MBNL1.

Having established the validity of the AP-FRET assay to detect intracellular interactions, we next used the technique to explore fluorescent fusion RNA-binding proteins. We chose proteins shown by *in vitro* binding assays to interact with the RNA foci in DM1 cells. Both CUGBP1 and hnRNP-C are distributed throughout the nucleoplasm and previous studies did not identify co-localization with RNA foci [19], but this does not preclude interactions that may not be detectable due to the high background signal from the nucleoplasmic signal. AP-FRET has the advantages of focusing on just the pixels where the RNA foci are and the proximity limits of FRET (i.e. 1–10 nm). Using our AP-FRET assay, it was clear that CUGBP1 and hnRNP-C do not interact with or were incapable of being detected in RNA foci with our assay (Fig. 3).

Our AP-FRET data also provides insights into the molecular basis of the RNA foci-MBNL1 interaction. Using deletion mutants of MBNL1, it is evident that all four zinc finger domains are necessary for this interaction (Fig. 4D). This is consistent with previous data from a three-hybrid assay which showed that all four ZF domains were necessary for strong interactions with CUG_21_ oligonucleotides [31], and more recently a study that showed that MBNL1 isoforms with all four ZF domains co-localized with RNA foci [40]. The *MBNL1* gene consists of at least 10 exons, and MBNL1 is expressed as many different alternatively spliced isoforms [34]. The isoform of *MBNL1* used in this study encodes exons 1 through 4, 6 and 10. The four zinc finger domains present in all isoforms are encoded by exon 1 and 2 (ZF1, ZF2) and exon 4 (ZF3, ZF4). Exon 3 encodes a “linker” domain connecting the two pairs of zinc fingers. It has been reported that this “linker” region was essential for the interaction of MBNL1 with CUG repeats based on three-hybrid assays [31]. In contrast, our AP-FRET assay shows that deletion of the “linker” region has negligible effects on the physical interaction between MBNL1 and RNA foci (Fig. 4C–D). The Del-C′ mutant shows that the carboxy terminus of MBNL1 including domains encoded by exon 6 and 10 are also dispensable for the interaction of MBNL1 to RNA foci.

Recently, the domains encoded by exons 3 and 6 were shown to be involved in the splicing of human c*TNT* and *IR* minigenes using co-transfection assays [40]. Though mini-genes are useful in mapping domains regulating splicing, they may not reflect intracellular behavior of the splicing targets. Instead, in our study we chose to look at endogenous splicing targets of MBNL1 that are implicated in DM1 pathology. Over-expression of MBNL1 in DM1 myoblasts rescued the splicing of *SERCA1* exon 22 and also increased exon 22 inclusion in HEK293T cells (Fig. 6A–B). In contrast, deletion of *MBNL1*-exon 3 (Del-Linker) or deletion of *MBNL1*-exon 6 (Del-C′) dramatically reduced the efficiency of MBNL1 in rescuing splicing defects in both DM1 myoblasts and the HEK293T cells (Fig. 6A–B). We also observed a similar effect in rescuing splicing defects for *NFIX* exon 7 (Fig. 6E). However, neither of these deletions resulted in a notable decrease in interactions with the RNA foci (Fig. 4C–D). In combination with the AP-FRET data, the results show that the ZF4 region of MBNL1 is important for both RNA foci interactions and regulation of alternative splicing. Both the Del-C′ and the Del-Linker constructs showed a significant reduction in splicing regulation for *SERCA1* and *NFIX* with no major change in RNA foci interaction which suggests that the domains required for MBNL1-RNA foci interaction can be different from the domains required for the regulation of alternative splicing events. The splicing targets analyzed in this study could be differentially regulated by MBNL1 and its mutants possibly by altering the recruitment or interactions of other splicing factors at the MBNL1-RNA splicing complex. RNA splicing requires the recruitment of multiple proteins to the splicing substrate. Recently, a number of RNA-binding proteins including hnRNPH, hnRNP-F and several others were identified as interacting partners of MBNL1 [41]. It may be that by deleting the sequences in MBNL1 encoded by exons 3 and/or 6, or even ZF4-C′ the recruitment of crucial components of the splicing machinery (i.e. other splicing factors) to the MBNL1-RNA complex is compromised, resulting in decreased splicing efficiency. The fact that *SERCA1* and *NFIX* splicing are more affected than *IR* exon 11 (Fig. 6C–D) suggests that different splicing targets are variably sensitive to these changes in stoichiometry.

FRET is one of the most sensitive techniques available for studying molecular interactions in cells and AP-FRET is an ideal method to investigate protein interactions in fixed cells. We have now developed and validated a novel FRET based assay for studying intracellular RNA-protein interactions. Furthermore, in validating this assay we have provided the first intracellular evidence of interaction between the mutant *DMPK* mRNA and MBNL1 and the mutant *DMPK* mRNA and hnRNPH. RNA foci have been found in a growing number of disorders including DM1, DM2, Fragile X Tremor and Ataxia Syndrome (FXTAS), Huntington Disease Like-2 (HDL-2), Spinocerebellar Ataxias (SCA8, SCA10) and more recently in Amyotrophic Lateral Sclerosis/Frontotemporal Dementia (ALS/FTD). Our study opens up new means of identifying and characterizing RNA-protein interactions in RNA foci complexes in such disease states and has the potential utility as an assay screening for compounds capable of disrupting deleterious RNA-protein interactions in DM1 and other disorders where these interactions play a key role in disease pathogenesis.
